# 血小板受体FcγRⅡA免疫学功能研究

**DOI:** 10.3760/cma.j.issn.0253-2727.2023.07.020

**Published:** 2023-07

**Authors:** 钊 张, 向慧 周, 志鹏 程, 豫 胡

**Affiliations:** 华中科技大学同济医学院附属协和医院血液科，武汉 430022 Department of Hematology, Union Hospital, Tongji Medical College, Huazhong University of Science and Technology, Wuhan 430022, China

血小板是来源于巨噬细胞的无核片段，广泛分布于人体血液循环中，其在生理性止血与病理性血栓形成中均发挥着重要作用[1]。近年来的研究表明，血小板在免疫应答及炎症反应中同样有着极其重要的作用[2]。血小板在免疫方面的作用包括病原体的识别、影响内皮屏障功能和改变血管通透性、释放储存的炎症因子、与白细胞相互作用并进行信号传导以及通过表面受体进行免疫调节[3]–[4]。近年来，针对血小板表面受体调节免疫信号的研究取得了明显进展，相关受体包括Toll样受体（TLR）、血小板糖蛋白Ⅵ（GPⅥ）、血小板Fc受体FcγRⅡA、钙离子依赖型凝集素样受体-2（CLEC-2）、血小板内皮黏附分子-1（PECAM-1）、癌胚抗原相关黏附分子-1（CEACAM-1）和髓样细胞触发受体样转录因子-1（TLT-1）等[5]。其中血小板表面受体FcγRⅡA是一种对单体免疫球蛋白（Ig）G具有低亲和力而对含IgG免疫复合物具有高亲和力的受体，无论是IgG单体还是免疫复合物中的IgG都通过Fc片段与FcγRⅡA结合。此外，FcγRⅡA也广泛分布在于巨噬细胞、中性粒细胞、部分树突细胞及其他细胞[6]，在血小板介导的各类免疫反应中发挥着重要作用。

一、血小板FcγRⅡA的结构及下游信号通路

FcγRⅡA是由FCGR2A基因编码的长约40 kDa的Ⅰ型跨膜蛋白，FCGR2A伴随着等位基因的变异（H/H131、H/R131和R/R131）[7]，这使得FcγRⅡA有多种表型。Fc受体由胞外区的两个Ig样结构域、跨膜区域和胞质尾部组成。FcγRⅡA胞外区第二个（靠近血小板膜）Ig样结构域介导与配体的结合，其尾部结构域含有免疫受体酪氨酸激活序列（ITAM）结构域，带有两个易于被磷酸化的酪氨酸（Tyr）残基（图1）。当胞外结构域与受体结合后，即可激活其尾部依赖ITAM的信号通路。值得一提的是，FcγRⅡA倾向于与配体体进行二聚结合，即2个FcγRⅡA与1个IgG免疫复合物进行结合，且这种结合的传导效果最佳。

**图1:**
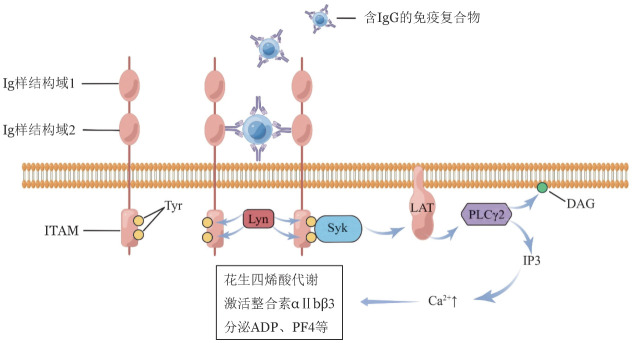
FcγRⅡA结构及下游信号通路 FcγRⅡA是结合IgG恒定区的恒定片段（Fc）γ受体家族成员，是一种Ⅰ型跨膜蛋白，由两个胞外Ig样结构域与胞内含免疫受体酪氨酸激活序列（ITAM）序列尾部组成。当FcγRⅡA与受体结合后，酪氨酸蛋白激酶（Lyn）磷酸化ITAM序列中的酪氨酸残基，随后募集并激活脾酪氨酸激酶（Syk），Syk启动T细胞活化衔接因子（LAT）信号，最终激活磷脂酶Cγ2（PLCγ2），生成甘油二酯（DAG）和三磷酸肌醇（IP3），引起Ca^2+^浓度升高，从而启动下游花生四烯酸代谢、整合素αⅡbβ3激活、二磷酸腺苷（ADP）与血小板因子4（PF4）分泌等过程

当FcγRⅡA与配体结合后引发Src家族激酶（SFK）与受体尾部对接，引起ITAM结构域中的YxxL序列中两个酪氨酸残基磷酸化。随后招募并激活含有SH2结构域的脾酪氨酸激酶（Syk）到受体尾部[8]，Syk激活T细胞活化衔接因子（LAT），LAT继续激活下游的磷脂酰肌醇-3-激酶（PI3KS）、磷脂酶Cγ2（PLCγ2），生成甘油二酯（DAG）和三磷酸肌醇（IP3）[7]，进而启动下游Ca^2+^动员、花生四烯酸代谢、脱颗粒、血小板因子4（PF4）释放和整合素αⅡbβ3激活等功能[9]–[11]（图1）。除FcγRⅡA外，血小板GPⅥ及CLEC-2受体也涉及ITAM依赖性的信号转导。GPⅥ是可结合胶原蛋白和纤维蛋白的血小板特异性受体，其尾部不含ITAM序列，但和含有ITAM序列的Fc受体c链形成复合物[12]。CLEC-2是血小板表面一种Ⅰ型跨膜糖蛋白，其尾部含ITAM序列[9]。

二、血小板FcγRⅡA在感染性疾病中的免疫学功能

机体外来病原微生物常常与各类血浆蛋白如各类抗体补体结合形成免疫复合物，而血小板FcγRⅡA对此类免疫复合物有较强亲和力，二者结合后即引发上文提及的信号通路从而发挥血小板各类免疫学功能。因此血小板FcγRⅡA在感染性疾病中发挥着极其重要的作用。

1. 细菌感染：越来越多的研究表明，许多细菌与血小板有着紧密而又复杂相互作用关系[13]–[14]。这种作用关系大体可分为三类。第一类是细菌与血浆蛋白结合后，结合细菌的血浆蛋白再与血小板结合。如金黄色葡萄球菌（简称金葡菌）通过血小板膜糖蛋白Ⅱb-Ⅲa（GPⅡb-Ⅲa）与血小板结合。血小板GPⅡb-Ⅲa是纤维蛋白原的受体，金葡菌表面分布着纤维蛋白原受体和纤维连接蛋白受体，包括凝集因子（Clf）A、ClfB、纤连蛋白结合蛋白A（FnbpA）和FnbpB。金葡菌通过上述受体结合纤维蛋白原后与血小板结合，而在这一过程中还需要IgG抗体与血小板FcγRⅡA的参与才能有效发挥血小板的功能。同样类似的还有金葡菌通过其表面蛋白A、血浆中的血管性血友病因子（VWF）、血小板GPⅠbα与血小板结合[15]。第二类是细菌直接与血小板进行结合。如表皮葡萄球菌表面的丝氨酸-天冬氨酸重复蛋白（SdrG），它不仅可以与纤维蛋白原结合，还可以直接结合血小板表面GPⅡb-Ⅲa。血链球菌表面的富含酪氨酸蛋白（Srp）A，可直接与血小板表面GPⅠbα结合。第三类是细菌分泌的产物与血小板结合。如厌氧菌牙龈卟啉单胞菌分泌的半胱氨酸蛋白酶，这一外毒素可以识别血小板蛋白酶激活受体（PAR）1并将其分解。金葡菌产生的葡萄球菌超抗原样蛋白（SLL）家族中的SLL5通过改变其残基结构与血小板GPⅠbα和GPⅣ相互作用。针对细菌的入侵，血小板可以分泌炎性因子募集免疫细胞同时释放如活性氧（ROS）、抗菌肽、防御素、蛋白酶等抗菌物质[16]–[17]。

在细菌诱导的血小板活化的过程中，FcγRⅡA起着类似枢纽的作用。在细菌通过多种特异性途径与血小板结合后，血小板活化的始动环节是FcγRⅡA与IgG和细菌组成的免疫复合物相结合[18]，而后引起FcγRⅡA的ITAM序列磷酸化激活上文提到的后续信号通路。值得注意的是，这一过程依赖于整合素αⅡbβ3。已经有研究证实在血小板缺乏αⅡbβ3或其被抑制的情况下，无法检测到FcγRⅡA的磷酸化以及后续的血小板分泌过程[18]–[19]。这反映了FcγRⅡA自身启动信号不足以激活下游通路，需要反馈信号协助。但整合素αⅡbβ3本身的参与并不会引起FcγRⅡA磷酸化，表现为在血小板激动剂诱导的血小板聚集中FcγRⅡA的磷酸化信号远低于细菌诱导[18]，这印证了细菌诱导的血小板活化的始动环节是FcγRⅡA与免疫复合物的结合。FcγRⅡA与αⅡbβ3协同作用激活下游信号通路，引起血小板分泌二磷酸腺苷（ADP）、血栓素（TXA）2和PF4。ADP与TXA2的作用是细菌诱导的依赖FcγRⅡA的血小板活化的又一协助信号。ADP与TXA2通过增强内源性αⅡbβ3的激活与α颗粒的释放形成对血小板活化的强级联正反馈信号[18]–[19]。αⅡbβ3、ADP与TXA2引起的反馈信号是FcγRⅡA介导的血小板活化成功的关键。

在血小板介导的抗菌过程中，FcγRⅡA同样发挥着重要作用。上文提到活化的血小板会释放PF4。PF4是一种带正电的四聚体蛋白，容易与带负电荷的聚阴离子结合，发生构象改变，暴露出新的抗原表位[20]。这些新的抗原表位会诱导免疫系统产生抗血小板因子4/肝素（anti-PF4/P）的IgG抗体[21]–[22]。PF4可以与多种细菌表面的聚阴离子结合（如革兰氏阴性菌脂多糖结构中的脂质A），从而引发抗PF4/P抗体结合。血小板FcγRⅡA通过识别经抗PF4/P的IgG抗体调理的细菌引起血小板对细菌的内化。这种内化不同于细胞吞噬，而是血小板通过对自身膜的扩展覆盖细菌，然后主动收缩，将细菌挤压到含抗菌物质的血小板颗粒附近[23]，这一过程除起到聚集细菌的作用外，还将细菌与体液隔离进而维持细菌周围抗菌物质的高浓度。这种对细菌的捕获过程依赖FcγRⅡA参与的血小板细胞骨架重构，即FcγRⅡA控制血小板中富含细胞骨架的动态丝状体与片状体进行移动、延伸[24]。此外，FcγRⅡA通过PF4介导的抗菌机制并不是血小板唯一的抗菌机制，已经有研究证明在体外环境下血小板可以独立于FcγRⅡA抑制金葡菌的生长，但这种情况下需要的血小板浓度要高于FcγRⅡA参与的情况[25]。

2. 病毒感染：病毒感染同血小板的关系同样密切，已经证实血小板减少、血栓形成、休克均与病毒感染有关[26]–[27]。在面对不同病毒入侵时，FcγRⅡA也起着不同的作用。在人类免疫缺陷病毒（HIV）感染的情况下，位于循环系统的病毒和非病毒成分大多以免疫复合物的形式存在，血小板FcγRⅡA对免疫复合物的结合与清除功效使得HIV的传染性降低。而在登革病毒（DENV）感染的情况下，机体针对DENV产生的抗体是疾病进展的主要因素之一，DENV与抗体形成免疫复合物后被FcγRⅡA识别后，DENV可经FcγRⅡA移动至宿主细胞内，促进DENV感染，这种机制同时会导致血小板减少并进展为登革出血热（DHF）[28]。在H1N1流感病毒感染中，H1N1病毒可以以免疫复合物的形式激活血小板引起血小板活化级联反应和花生四烯酸代谢[29]，并引起血小板分泌多种炎症因子。这一过程既有利于机体调动免疫细胞抵抗病毒感染，又可以产生如血清素等介导免疫复合物驱动性休克的物质[30]。在H1N1流感病毒感染中仍然可以观察到血小板减少症的出现，可能的一种解释为含有病毒的免疫复合物可以诱导血小板分解为血小板微粒或被网状内皮系统清除[29]。最新研究证实，FcγRⅡA在新型冠状病毒感染引发的血小板过度活化中起着关键作用。在经过抗体介导的中和作用、耗竭IgG，应用Syk抑制剂阻断血小板FcγRⅡA后，有效改善了新型冠状病毒引发的血小板过度活化以及内皮微流腔条件下的血小板聚集[31]。

3. 真菌感染：虽然有研究提出血小板释放的抗菌肽可以对真菌产生杀伤作用，但这种抗真菌效果在最近的实验中并没有再现[32]，血小板的抗真菌作用仍然存疑。目前可以确定的是真菌能够引起血小板活化，并且FcγRⅡA参与其中。有学者证实了卷枝毛霉NRRL3631通过FcγRⅡA与整合素αⅡbβ3引起血小板活化、聚集[33]，并且受SFK、Syk及次级介质TXA2、ADP的调控[33]。需要注意的是真菌引发血小板活化的方式根据其物种而异，如白色念珠菌通过GPⅡb/Ⅲa而非FcγRⅡA引起血小板活化[34]。真菌通过血小板表面受体介导的血小板活化提示了血小板表面受体在真菌感染中引发的血栓形成中的作用。血小板表面受体有成为真菌引起的血栓治疗靶点的可能。

4. 寄生虫感染：目前已知血吸虫与锥虫可与血小板相互作用，体外动物实验证实两种寄生虫均会导致不同程度的血小板减少症[35]。当前研究最多的与血小板相互作用的寄生虫为疟原虫，疟原虫感染的常见特征同样为血小板减少症[36]，同时血小板减少也可以作为疟原虫感染可疑患者的诊断指标以及确诊患者的预后参考[37]。血小板FcγRⅡA似乎参与了疟原虫与血小板的相互作用。一项证据是疟疾患者体内血小板IgG水平升高，并显示出血小板活化的迹象，这表明疟原虫引发的血小板减少可能与免疫功能相关[37]。另一项证据是虽然并无动物实验证实FcγRⅡA在疟原虫感染中发挥着作用，但携带FcγRⅡA-H131基因型的人群对疟原虫有着更高的易感性[38]。这表明血小板FcγRⅡA在疟原虫感染中发挥着未知的作用。

三、血小板FcγRⅡA在非感染性疾病中的免疫学功能

在一些非感染性疾病如自身免疫性疾病与肿瘤性疾病中，经常发生炎症反应过度激活、免疫系统失调、组织损伤等过程，这些过程往往与血小板FcγRⅡA的作用有关。

1. 肝素诱导的血小板减少症：由于普通肝素（UFH）在各类心血管手术中有着疗效好、起效快等优势，使其在临床中无法被取代。然而在UFH使用的过程中要警惕肝素诱导的血小板减少症（HIT）的发生。HIT是由结合了IgG的PF4与肝素结合成为免疫复合物后由FcγRⅡA介导的疾病，其特点是显著的血小板减少并伴随血栓形成的风险[39]。作为阳离子四聚体蛋白的PF4与作为肝素的阴离子化合物通过电荷作用相互结合，这一过程既稳定了PF4四聚体构象，又使PF4与肝素更易结合PF4抗体形成超大免疫复合物（ULIC）[40]。ULIC随后与血小板表面的FcγRⅡA结合引发ITAM下游信号通路，这引起血小板表面P选择素的上调进而促进血栓形成或血栓前状态的形成。血小板下游信号通路的激活同时会引起PF4的释放，反过来加速ULIC的生成。有研究表明FcγRⅡA-R131等位基因在HIT的患者中过度表达，明显增加了血栓形成的风险[41]。

2. 系统性红斑狼疮（SLE）：SLE是一类自身产生抗双链DNA抗体（抗dsDNA抗体）、抗核抗体与抗磷脂抗体并进一步结合为免疫复合物导致组织损伤的疾病。其特点是过度炎症反应、血小板减少，血栓形成以及易引起肾脏疾病[42]。血小板在SLE的发生发展中起着不可或缺的作用，表现为SLE的免疫功能紊乱引起血小板活化、血栓形成、血小板减少、激活其他免疫细胞而加剧免疫失调[43]。血小板过度活化可能是由血小板FcγRⅡA介导的，已经有研究证实SLE相关IgG或免疫复合物在体外可通过FcγRⅡA激活血小板[44]。值得一提的是，编码FcγRⅡA的FCGR2A不同等位基因同样影响着SLE的易感性和预后。在SLE患者与正常人群的对比中，FcγRⅡA-R131基因型的比例显著增高[45]，同时FcγRⅡA-R131基因型的患者更容易发生血小板减少症。此外，有学者在FcγRⅡA转基因SLE小鼠模型中观察到了肺部和肾脏的血栓形成并加重了狼疮肾炎[11]。

3. 肿瘤：血小板在肿瘤的生长、转移以及血管形成中发挥着重要作用[46]–[47]。进入血液循环中的肿瘤细胞想要到达其他部位形成局部转移灶，需要克服血液循环中的高剪切力并逃离免疫系统的监视。肿瘤细胞释放的一些激活血小板的可溶性介质（ADP、TXA2等）与血小板表面TLR4结合引起血小板活化[48]。局部的血小板活化促进了血栓形成，并将肿瘤细胞包裹在血栓内防止被免疫系统清除，同时这也是肿瘤患者血液高凝易引发血栓的原因之一。最近有学者利用免疫共沉淀证实血小板FcγRⅡA通过与癌细胞衍生的IgG相互作用引起血小板活化，并且观察到肝癌患者血小板表面FcγRⅡA比例上调[49]。除此之外，肿瘤细胞还有着诱导血小板分泌的能力。肿瘤细胞诱导的血小板分泌（TCIPS）可产生多种有利于肿瘤生长转移的因子，如转化生长因子-β（TGF-β）和血管内皮生长因子（VEGF），这些因子促进了肿瘤转移及肿瘤内部血管形成。有研究证实Syk、磷脂酶C（PLC）、αⅡbβ3的抑制剂可有效阻滞TCIPS过程[50]，值得注意的是他们都参与血小板免疫相关受体ITAM下游信号通路。在利用抗原结合片段（Fab）抗体IV.3抑制血小板FcγRⅡA后，血小板的分泌与聚集基本被阻断[50]。这些都说明血小板FcγRⅡA在肿瘤细胞介导的血小板活化与分泌中发挥着不可或缺的作用，其有望成为肿瘤治疗干预中的重要靶点。

4. 疫苗诱导的血栓性血小板减少症：疫苗诱导的血栓性血小板减少症（VITT）是一类罕见而严重的疾病，是由接种新型冠状病毒腺病毒载体疫苗-ChAdOx1 nCOV-19（澳大利亚悉尼AstraZeneca公司）和Ad26.COV2.S（澳大利亚悉尼Janssen/Johnson公司）引起的综合征，其特征是血小板减少、血栓形成、D-二聚体升高、抗PF4抗体生成[51]–[52]。接种第一剂疫苗的患病率约为2/10万，第二剂患病率极低[51]。VITT的发病机制与HIT类似，患者体内的抗PF4抗体参与形成免疫复合物后，与FcγRⅡA交联而引起广泛的血小板活化[53]。这一过程促进了凝血酶的生成，表现为血小板减少与血栓形成。在HIT中，带正电的PF4与带负电的肝素结合产生抗原表位引起抗PF4抗体生成。而在VITT中，与带正电的PF4结合的物质有两种假设。首先考虑为疫苗中带负电的腺病毒载体与PF4结合[54]，这种情况需要疫苗颗粒大量进入血液，因而这一假设可能性较低[55]。第二种假设为疫苗产生的刺突蛋白和相关炎症反应诱导了内皮损伤和糖胺聚糖的脱落，最终导致PF4与内源性糖胺聚糖结合[56]。当前针对VITT的一线疗法是静脉注射免疫球蛋白（IVIg）预防FcγRⅡA介导的血小板活化来改善血液高凝状态[57]。有学者提出利用治疗B淋巴细胞恶性肿瘤的布鲁顿酪氨酸激酶（Btk）抑制剂来阻断FcγRⅡA介导的Btk下游信号通路，从而抑制FcγRⅡA交联引起的血小板聚集、P选择素表达、致密颗粒分泌过程[58]。但Btk的使用将抑制患者依赖血小板FcγRⅡA与αⅡbβ3的抗菌功能，增大了患者感染风险[59]。临床上应对患者状况进行综合评估后谨慎使用。

四、结语

由于血小板识别并杀伤病原微生物、释放各类炎症因子及免疫调节剂，炎症期间维持血管完整性等功能，血小板已经被确定为免疫系统重要组成部分。无论是在感染性疾病还是非感染性疾病中，FcγRⅡA在血小板介导的免疫学功能中发挥着不可或缺的作用。FcγRⅡA是血小板识别某些病原微生物的枢纽，同时又参与杀伤某些微生物的始动环节。然而其介导的免疫学功能是一把双刃剑，虽然在机体对抗某些病原微生物过程中起着正向积极作用，但加剧了某些感染性疾病的病情发展，同时也引起了一些疾病中血小板减少与病理性血栓的形成。当前我们对血小板FcγRⅡA的免疫功能仅有初步的认识，虽然有大量证据证实FcγRⅡA有着重要的免疫学功能，但目前临床上缺乏将其作为治疗靶点的药物，而且其生理性功能仍有待发掘。继续深入研究血小板FcγRⅡA对了解血小板免疫功能、防治各种免疫相关血小板疾病具有重大意义。
